# Mitigation Technique for Receiver Performance Variation of Multi-Color Channels in Visible Light Communication

**DOI:** 10.3390/s110606131

**Published:** 2011-06-07

**Authors:** Muhammad Shahin Uddin, Jae Sang Cha, Jin Young Kim, Yeong Min Jang

**Affiliations:** 1 Department of Electronics Engineering, Kookmin University, Seoul, Korea; E-Mail: shahin.mbstu@gmail.com; 2 Department of Media Engineering, Seoul National University of Science & Technology, Korea; E-Mail: chajs@snut.ac.kr; 3 Department of Wireless Communications Engineering, Kangwoon University, Korea; E-Mail: jinyoung@kw.ac.kr

**Keywords:** multi-color channels, visible light communication, photo sensitivity, photo-detector, outage probability, performance balancing factor

## Abstract

“Green” and energy-efficient wireless communication schemes have recently experienced rapid development and garnered much interest. One such scheme is visible light communication (VLC) which is being touted as one of the next generation wireless communication systems. VLC allows communication using multi-color channels that provide high data rates and illumination simultaneously. Even though VLC has many advantageous features compared with RF technologies, including visibility, ubiquitousness, high speed, high security, harmlessness for the human body and freedom of RF interference, it suffers from some problems on the receiver side, one of them being photo sensitivity dissimilarity of the receiver. The photo sensitivity characteristics of a VLC receiver such as Si photo-detector depend on the wavelength variation. The performance of the VLC receiver is not uniform towards all channel colors, but it is desirable for receivers to have the same performance on each color channel. In this paper, we propose a mitigation technique for reducing the performance variation of the receiver on multi-color channels. We show received power, SNR, BER, output current, and outage probability in our simulation for different color channels. Simulation results show that, the proposed scheme can reduce the performance variation of the VLC receiver on multi-color channels.

## 1. Introduction

Nowadays Light Emitting Diodes (LEDs) are considered to represent the next generation lighting and communication devices. LEDs have some tremendous features, such as small size, low power consumption, long life, fast response times, and low cost. LEDs are used in numerous applications, such as in color displays, traffic signals, sign boards, automobiles, LED TV, and cellular phones *etc*. In visible light communication (VLC), LEDs are used both as communication transmitters and lighting devices. A VLC system is a type of optical wireless communication system in which visible light is used as a transmission medium. This is safe for the humans because it functions in the visible spectrum, and provides a high rate of data transmission [1]. Due to the high energy efficiency of the LED, a high optical signal to noise ratio is achieved using only a few watts of power. Compared with radiowave wireless communication, VLC is harmless to humans, provides high security, is license free and does not cause the malfunction of aircraft equipment or medical instruments [1]. Among the many applications using colored light sources that can be considered in VLC are: (i) the scenario in which a VLC receiver receives some information from traffic signal light sources with color “A” and color “B” or (ii) a VLC receiver receives audio information from a color “A”, video information from a color “B” and navigation information from a color “C” or (iii) multiplexing technologies such as Wavelength Division Multiplexing (WDM) can be applied to VLC applications using the colors “A”, “B” and “C” [2,3]. Most of these cases will require VLC services using multiple color channels according to the VLC band plan. A typical VLC receiver may display high receiver performance only on, for example, the color “A” channel but it doesn’t show the same performance on the color “B” or color “C” channels [2]. It may be desirable for a VLC receiver to have the same performance on each color channel because users may want the performance of the receiver to be maintained uniformly on every color channel, according to the VLC band plan. However, there are two main factors influencing the performance variation of a multi-color VLC receiver. One is the conversion relations between the radiometric and photometric units when the received signal power going into a receiver is defined in VLC. The performance variation of a VLC receiver according to multiple color channels is often due to the fact that the photo sensitivity characteristics of a photo-detector such as Si photo-detector (assuming such photo-detectors will be used as a receiver in VLC) depends on the wavelength variation. The photo sensitivity value of a Si photo-detector is higher for the longer wavelengths than for the shorter wavelengths in the visible band. Si photo-detectors thus produce more electrical current on the red color channel than on green or blue color channel, even though the radiometric received powers on each color channel are equal. Eventually a VLC receiver with Si photo-detector reacts differently on multiple color channels, even though the radiometric received powers are equal on each color channel [2]. Therefore, one of the main factors, the dependency of the photo sensitivity of a photo-detector on wavelength, needs to be considered in order to ensure that the performance of a VLC receiver can be maintained uniformly on multi-color channels. Many researchers have proposed VLC systems for improving indoor and outdoor performance, but no one has considered the performance variation due to different color of channels. Lee *et al.* have proposed a receiver structure to improve the VLC system where separate receivers with specific spectral response are used for the detection of different colors of channels but they did not consider the performance variations of the receiver on a different color of channels. We therefore propose a receiver structure in order to reduce the problem of performance variation according to the multi-color channels and maintain the receiver output current uniform as much as possible for all color of channels.

The remainder of this paper is organized as follows: Section 2 introduces the proposed system model; In Section 3 the SNR, BER, and Outage probability expressions are derived; Results are presented and discussed in Section 4 before we conclude this paper in Section 5.

## 2. System Model

**Figure 1.**
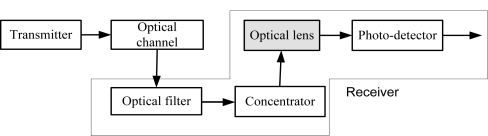
System models with proposed receiver structure.

LEDs are used to transmit desired optical signal in visible light communication system. The desired optical signals then travel through air before reaching VLC receiver. The receiver collects some undesirable optical signals which cause severe degradation to the overall system performance. Optical filter is used to minimize the background noise. In visible light communication system receiver performance variation is occurred due to the wavelength variation on multi-color channel. In our proposed system convex lens with different refractive index for different wavelength are used to mitigate the performance variation of the receiver. Our system model is shown in Figure 1.

### 2.1. Transmitter Model

Visible light links are commonly classified according to two criteria, namely, the degree of directionality of the transceiver and the link relies upon the existence of a line-of-sight (LOS) path between them. The line of sight links employ narrow field of view (FOV) transceivers that must be aimed in order to establish a communication link, while non-line of sight links employ wide FOV transceivers that obviate the need for such positioning. LOS links rely upon a direct path between the transmitter and receiver for communication, whereas non-LOS links usually rely upon reflection of the light from the ceiling or some other diffusely reflecting surface [4–6]. In general, LOS links minimize path loss and maximize the power efficiency, and they can achieve higher transmission rates, however, they are less robust and less convenient to use. NLOS links increase robustness and ease of use, allowing high user mobility and the links to operate even when there are barriers between the transmitter and the receiver but they suffer from lower transmission rates. For better performance in the case of NLOS link one has to consider a higher degree of reflection.

### 2.2. Channel Model

**Figure 2.**
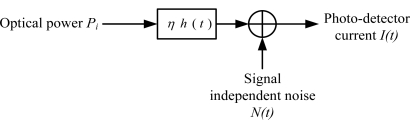
Modeling of VLC channel with IM/DD.

For a low cost visible light communication system, the most viable modulation is the intensity modulation (IM), in which the desired waveform is modulated onto the instantaneous power of the carrier. On the other hand, the most practical down-conversion technique is the direct detection (DD), in which a photo-detector produces current proportional to the received instantaneous power [7–9]. Figure 2 shows the modeling of visible light communication channels with IM/DD.

Modeling a visible light communication link as a baseband linear, time-invariant system having impulse response *h*(*t*), with signal-independent additive noise *N*(*t*), the visible-light channel is modeled as a linear optical additive white Gaussian noise (AWGN) channel and summarized by the following expression [10]:

(1) $$I(t)=\eta P_{i}(t)⊗h(t)+N(t)$$

where *I*(*t*) is the photo-detector current, *η* represents the photo sensitivity of the photo-detector (in A/W), *P_i_*(*t*) is the instantaneous input power, the symbol “⊗” denotes convolution, *h*(*t*) resembles the impulse response and *N*(*t*) is the AWGN. The time average transmitted optical power *P_t_* is given by [10]:

(2) $$P_{t}={\text{lim}}_{T\to \infty }\frac{1}{2T}\int_{-T}^{T}P_{i}(t)dt$$

where *P_i_*(*t*) ≥ 0 since the channel input power must be nonnegative.

The average received optical power *P_r_* generally can then be determined by:

(3) $$P_{r}=H(0)P_{t}$$

where $H(0)=\int_{-\infty }^{\infty }h(t)dt$ is the channel DC gain.

In this paper consider line-of-sight (LOS) and non line- of- sight (NLOS) links are considered.

#### 2.2.1. LOS Case

In line of sight case the received power is generally determined by:

(4) $$P_{rLOS}=H_{LOS}(0)P_{t}$$

#### 2.2.2. NLOS Case with LOS

In the NLOS case, let us consider the effect of light reflected by walls or other obstacles. The received power is generally given by the channel DC gain on LOS and reflected path *H_ref_*(0):

(5) $$\begin{array}{lll} P_{r} & = & H_{LOS}(0)P_{t}+H_{NLOS}(0)P_{t}\\ & = & H_{LOS}(0)P_{t}+\sum_{ref}H_{ref}(0)P_{t} \end{array}$$

### 2.3. Receiver Model

**Figure 3.**
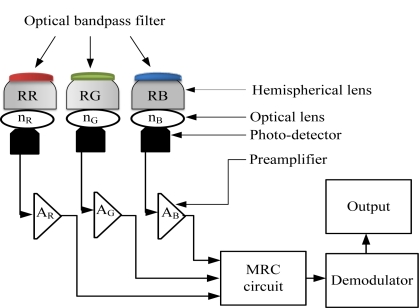
Proposed receiver structures.

We propose a receiver structure as shown in Figure 3, which comprises three receiving front-ends and employs the combining technique, in order to effectively suppress the ambient noise and allow optimum detection of the desired optical signals. Each receiving front-end is constructed from an optical filter, optical concentrator, optical lens, photo-detector and preamplifier. We consider the use of optical bandpass filters in our proposed design to allow significant reduction of the ambient light noise. It is assumed that the bandpass filters have maximum signal transmission within their optical passband regions. We employ a non-imaging hemispherical optical concentrator to achieve larger effective signal collection area, wider FOV and omnidirectional gain. The bandpass filter is deposited onto the outer surface of the hemispherical concentrator to minimize the shift in the filter passband and maximize its transmission [10]. An optical lens with a different refractive index for different color bands is attached to the inner surface of the hemispherical optical concentrator, and then a silicon photo-detector with fast switching capability is attached. The response of the photodiode varies with visible-light signals at different spectral wavelengths and it produces different output currents even though the input optical power is the same. Our proposed receiver includes three lenses with different refractive indexes for the red, green and blue color band. We assume the use of a low noise field-effect transistor (FET)-based transimpedance preamplifier to achieve a large dynamic range and a wide bandwidth. All of the receiving branches are connected to a maximal ratio combining (MRC) circuit to achieve highest SNR that is connected to the output. The output requires further signal processing to restore the desired waveform.

## 3. Theoretical Analysis

A larger signal detection area is desired to improve the overall performance of the VLC system. However, increasing the photodiode area incurs more cost to the design, and tends to decrease the receiver bandwidth and increase the receiver noise. As an alternative to achieve a larger effective signal collection area, we employ a non-imaging hemispherical optical concentrator with concentrator radius R, concentrator FOV *ψ_c_*, and internal refractive index *n*. With a hemispherical filter, our receiver could achieve a narrow bandwidth and wide FOV simultaneously. The receiver structure consists of three receiving front-ends for capturing the red, green and blue signals from the LED transmitter. The proposed design employs optical band pass filters with narrower bandwidths of *Δλ*_R_, *Δλ_G_* and *Δλ_B_* and optical lens with refractive index of *n_R_*, *n_G_*, and *n_B_* in receivers *RR*, *RG* and *RB*, respectively. Here *RR*, *RG* and *RB* means receiver for red color, receiver for green color and receiver for blue color, respectively. Receiver *RR* with an optical bandwidth between *λ*_*R*1_ and *λ*_*R*2_ allows the red signals to pass through, receiver *RG* with an optical bandwidth between *λ*_*G*1_ and *λ*_*G*2_ allows the green signals to pass through and receiver *RB* with an optical bandwidth between *λ*_*B*1_ and *λ*_*B*2_ allows the blue signals to pass through. Consequently, all of the receiving branches are connected to a maximal ratio combining (MRC) circuit. The output of the MRC circuit requires further signal processing to restore the desired waveform. We consider the band pass filters to have maximum signal transmission within their optical pass band regions. In addition, it is assumed that the silicon photodiodes and the FET-based trans-impedance preamplifiers used in all receivers share similar characteristics. To increase the separation distance between a light transmitter and a receiver, lenses are often used. A light receiver may use a lens to collect the weak light from the transmitter and focus it onto the receiver’s detector for processing. But, the lens will always collect extra light from the environment that is not wanted. Stray light will often interfere with the signals of interest [7–9]. In our proposed structure we use band pass filter before lens.

Our proposed receiver structure received power is given by the following equation:

(6) $$P_{r}=\zeta (n_{lens}(\lambda ))H(0)P_{t}$$

where *ζ*(*n_lens_*(*λ*)) is the performance variation balancing factor:

(7) $$\begin{array}{cc} \text{and}\;\text{where}: & \zeta (n_{lens}(\lambda ))=\frac{P_{olens}(n_{lens}(\lambda ))}{P_{ilens}}\\ \text{where}: & P_{olens}=\frac{\left(n_{lens}(\lambda )-n_{0}\right)}{n_{0}}\left(\frac{1}{R_{1}}-\frac{1}{R_{2}}\right)\\ & P_{ilens}=H(0)P_{t},\;\text{and}\;\lambda \in (\lambda_{R},\lambda_{G},\lambda_{B})\\ & n_{lense}(\lambda_{R})=n_{R},\;\;n_{lens}(\lambda_{G})=n_{G},\;\text{and}\;n_{lens}(\lambda_{B})=n_{B} \end{array}$$

*P_items_* and *P_olens_* are the input and output power of the lens respectively, *n_lens_*(*λ*) and *n_0_* are the refractive index of the lens and air respectively, *R*_1_ and *R*_2_ are the radius of the lens. Optical received power is different for different optical communication links [11,12]. Here we consider two types of link, one is line-of-sight (LOS) and another one is non line-of-sight (NLOS).

### 3.1. LOS Case

In line of sight case the received power is determined by:

(8) $$P_{rLOS}=\zeta (n_{lens})H_{LOS}(0)P_{t}$$

For convenience we use *ζ*(*n_lens_*) instead of *ζ*(*n_lens_*(*λ*))

The channel DC gain *H_LOS_* can be determined by the following expression [10]:

(9) $$H_{LOS}(0)=\{\begin{array}{ll} \frac{(m+1)A}{2\pi D^{2}}{\text{cos}}^{m}(\varphi )T_{s}(\psi )g(\psi )\text{cos}(\psi ) & 0\leq \psi \leq \psi_{c}\\ 0 & elsewhere \end{array}$$

where *m* is the order of Lambertian emission, *A* is the photo-detector area, *D* is the distance between transmitter and receiver, *ϕ* is the angle of irradiance, *ψ* is the angle of incidence, *T_s_*(*ψ*) is the signal transmission coefficient of an optical filter, *g*(*ψ*) is the gain of an optical concentrator, and *ψ_c_* is the receiver field of view (FOV) [12]. The order of Lambertian emission *m* can be found from the equation [13], $m=-\frac{\text{ln}2}{\text{ln}(\text{cos}\Phi_{1/2})}$ where *Φ*_1/2_ is the transmitter half power angle. The gain can be determined from the following expression [9]:

(10) $$g(\psi )=\{\begin{array}{ll} \frac{n^{2}}{{\text{sin}}^{2}\psi_{c}} & 0\leq \psi \leq \psi_{c}\\ 0 & elsewhere \end{array}$$

where *n* denotes the internal refractive index of the optical concentrator.

### 3.2. NLOS Case with LOS

In NLOS case, let us consider the effect of reflected light by walls or other obstacles. The received power is given by the channel DC gain on LOS and reflected path and reflected path H_ref_(0):

(11) $$P_{r}=\zeta ({\text{n}}_{\text{lens}})H_{LOS}(0)P_{t}+\zeta ({\text{n}}_{\text{lens}})\sum_{ref}H_{ref}(0)P_{t}$$

**Figure 4.**
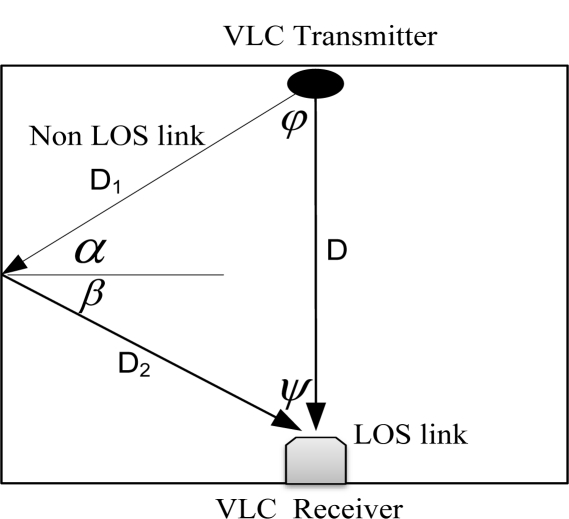
VLC transmitter model with LOS and NLOS link.

The DC gain on the first reflection is [10]:

(12) $$H_{ref}(0)=\{\begin{array}{ll} \frac{(m+1)A}{2\pi D_{1}^{2}D_{2}^{2}}\xi dA\;{\text{cos}}^{m}(\varphi )\text{cos}(\alpha )\text{cos}(\beta )T_{s}(\psi )g(\psi )\text{cos}(\psi ) & 0\leq \psi \leq \psi_{c}\\ 0 & elsewhere \end{array}$$

where *D*_1_ is the distance between transmitter and reflective point, *D*_2_ is the is the distance between reflective point and receiver, *ζ* is the is the reflectance factor, *dA* is reflective area of small region, *φ* is the angle of irradiance to a reflective point, *α* is the angle of irradiance to the receiver, *β* is the angle of incidence to the receiver, *ψ* is the angle of incidence as shown in Figure 4.

### 3.3. Signal-to-Noise Ratio (SNR)

A SNR can express the quality of a communication system. We assume that the transmitter transmits the signal using on-off keying (OOK) modulation technique. Among all modulation techniques for visible light communication link, OOK is the simplest one and it is very easy to implement. In a single receiver, the average signal-to-noise ratio (SNR) is defined as the ratio of the received signal to the aggregated noise and it can be seen that when the shot noise is the dominant noise source, the SNR is proportional to the detector area [12]. The signal component of the signal to noise ratio is measured by:

(13) $$S=\eta^{2}\zeta^{2}({\text{n}}_{\text{lens}})P_{rSignal}^{2}$$

where desired signal power *P_rSignal_* is:

(14) $$P_{rSignal}=\int_{0}^{T}\left(\sum h_{i}(t)⊗P_{i}(t)\right)dt$$

Further, multipath fading can be neglected in optical wireless channel. In our channel model, the information carrier is a light wave. Moreover, detector dimensions are in the order of thousands of wavelengths, leading to efficient spatial diversity, which prevents multipath fading. For the above reasons, multipath fading can be neglected [10]. We assume OOK with rectangular transmitted pulses of duration equal to the bit period. Gaussian noise having a total variance *N* that is the sum of contributions from shot noise, thermal noise and intersymbol interference by an optical path difference:

(15) $$N=\sigma_{shot}^{2}+\sigma_{thermal}^{2}+\eta^{2}P_{rISI}^{2}$$

Therefore the signal to noise ratio is given by:

(16) $$SNR=\frac{\eta^{2}\zeta^{2}(n_{lens})H^{2}(0)P_{t}^{2}}{\sigma_{shot}^{2}+\sigma_{thermal}^{2}+\eta^{2}P_{rISI}^{2}}$$

and BER is given by:

(17) $$BER=Q(\sqrt{SNR})$$

where:

(18) $$Q(x)=\frac{1}{2\pi }\int_{x}^{\infty }e^{-y^{2}/2}dy$$

The received power by inter-symbol interference *P_rISI_* is given by:

(19) $$P_{rISI}=\int_{T}^{\infty }\left(\sum h_{i}(t)⊗P_{i}(t)\right)dt$$

Shot noise variance is given by:

(20) $$\sigma_{shot}^{2}=2q\eta (P_{rSignal}+P_{rISI})B_{en}+2qI_{bg}I_{2}B_{en}$$

where *P_rSignal_* is the received power, *P_rISI_* is the received power by inter-symbol intereference, q is the electronic charge, *B_en_* is equivalent noise bandwidth, *I_bg_* is background current and I_2_(= 0.562) is noise bandwidth factor.

The thermal noise variance is given by:

(21) $$\sigma_{thermal}^{2}=\frac{8\pi kT_{k}}{G}\delta AI_{2}B_{en}^{2}+\frac{16\pi^{2}kT_{k}E}{g_{m}}\delta^{2}A^{2}I_{3}B_{en}^{3}$$

where *k* is Boltzmann’s constant, *T_k_* is absolute temperature, *G* is the open-loop voltage gain, *δ* is the fixed capacitance of photo-detector per unit area, *E* is the FET channel noise factor, g_m_ is the FET transconductance, and *I*_3_ = 0.868 [10].

### 3.4. Outage Probability

The outage probability of a system is the probability that the instantaneous signal-to-noise ratio (SNR) falls below a specified threshold *Γ_th_* and denoted as [14,15]:

(22) $$\begin{array}{lll} P_{out} & = & P_{r}(SNR<\Gamma_{th})\\ & = & P_{r}\left(\frac{\eta^{2}Y^{2}}{\sigma_{shot}^{2}+\sigma_{thermal}^{2}+\eta^{2}P_{rISI}^{2}}<\Gamma_{th}\right)\\ & = & P_{r}\left(Y^{2}<\frac{\sigma_{shot}^{2}+\sigma_{thermal}^{2}+\eta^{2}P_{rISI}^{2}}{\eta^{2}}<\Gamma_{th}\right) \end{array}$$

where *Y* = *f*{*λ ∈* (*λ_R_*, *λ_G_*, *λ_B_*), *FOV*, *ζ*} is a random variable of desired signal.

In our system model outage probability depends upon the performance variation balancing factor *ζ* and the instantaneous received power. The receiver power relies on the DC gain when the transmitted power is fixed. DC gain is the function of receiver FOV. SNR changes significantly with the change of the FOV of the receiver. When FOV increases then the SNR decreases, on the other hand SNR increases if *ζ* increases. Therefore the outage probability of our system is given by the following equation:

(23) $$P_{out}(\lambda_{x},FOV)=\int_{0}^{\frac{\sigma_{shot}^{2}+\sigma_{thermal}^{2}+\eta^{2}P_{rISI}^{2}}{\eta^{2}}\Gamma_{\text{th}}}\frac{1}{\zeta^{2}(\lambda_{x})}e^{-Y^{2}(FOV)}dY$$

where *λ_x_* is the wavelength of different color of channels. Here only red, green and blue color channels are considered.

## 4. Results and Discussion

We compare the variations of receiver power, SNR, BER, outage probability and output current between the traditional scheme and the proposed scheme. Simulations are done with the MATLAB software. In a visible light communication system every receiver expects same performance, even though the receiver uses different color of channel. Therefore our scheme focuses on reducing the performance variation at the receiver side. In our analysis, we consider three channel colors such as red, green and blue. All outputs show that the performance variation of the VLC receiver is reduced by using our proposed scheme. The following parameters are considered in Table 1 for finding the results.

**Figure 5.**
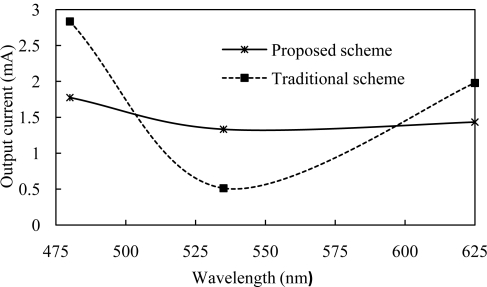
Photo-detector output current *vs*. wavelength.

Figure 5 shows the output current for both traditional and proposed VLC receiver photo-detector at the center wavelength 480 nm, 535 nm and 625 nm. Here output current variation under the same transmitter and receiver is reduced by using the proposed scheme.

**Figure 6.**
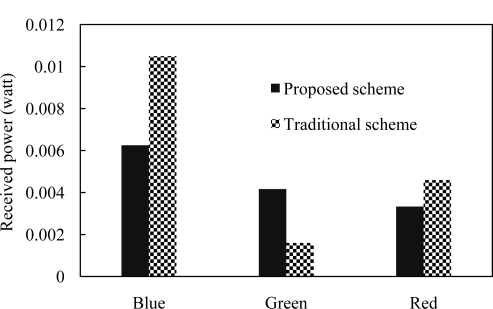
Varied received power (in watt) with different color channels.

Figure 6 shows the received power for both traditional and proposed VLC receiver at different color of channels. Received powers for blue, green and red color channels are not constant. But this variation affects the performance of the system even though receiver expects same performance for each color of channel. Our proposed scheme reduces the variation of received power and improves the performance as well.

**Figure 7.**
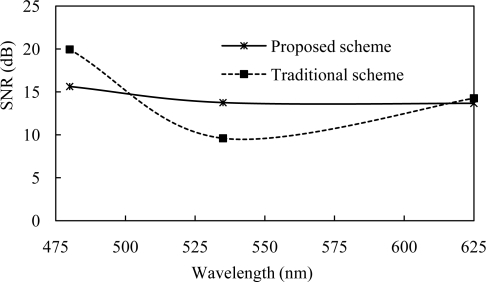
Received SNR *vs*. wavelength.

Figure 7 shows the SNR for both traditional and proposed VLC receiver at the center wavelength of different color of channels. SNR is directly proportional to the square of instantaneous received power. SNR variation affects the data rate of the VLC system. SNR also depends upon multipath fading, inter-symbol interference and other noise. Our proposed scheme reduces the variation of the SNR on red green and blue color of channels.

**Figure 8.**
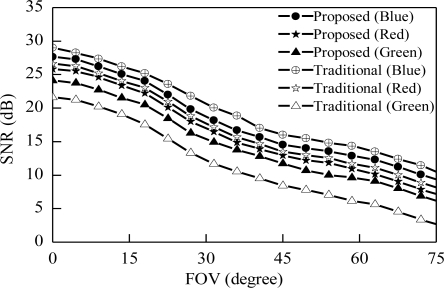
Received SNR *vs*. FOV.

Figure 8 shows the relation between FOV and received SNR. In a visible light communication system FOV of the receiver is important for high data rates. We plot here proposed and traditional receiver SNR variation with varying the FOV of the receiver. Figure 8 shows that the variation of SNR with multi-color channels. SNR variation of traditional scheme from minimum to maximum depending on the color is larger than proposed receiver system. At FOV 30 degree received SNR for green, red and blue color channel are 12.5 dB, 17.5 dB and 22 dB respectively in traditional case. The SNR variation between minimum to maximum is 9.5 dB but our proposed case this variation is only 3 dB. This result indicates that our proposed scheme reduces the SNR variation of the receiver.

**Figure 9.**
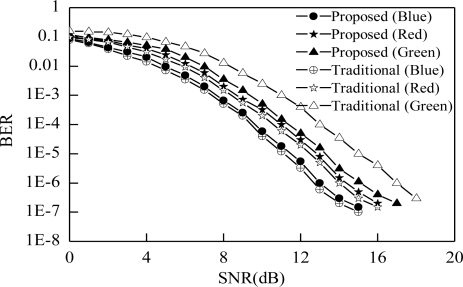
BER *vs*. Received SNR.

Figure 9 shows the BER performance of both the proposed and traditional color channel. Different channel colors have different BER performance. BER of the green color channel is reduced using the proposed scheme even though other color BER is increased a little bit but the variation of BER performance among the different colors is reduced more. Therefore we can state that the performance variation due to multi-color channel is reduced by using the proposed scheme.

**Figure 10.**
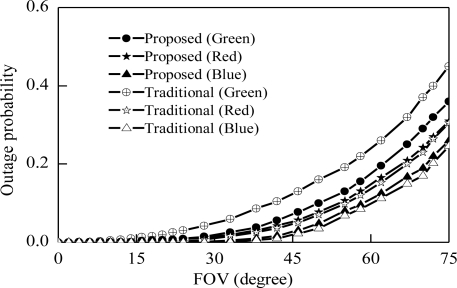
Outage probabilities *vs*. FOV.

Figure 10 shows the relation between outage probability and receiver FOV at each color of channel for both proposed and traditional scheme. This Figure shows that outage probabilities for different colors are different. The variation among the colors is a severe problem in visible light communication systems. Green color channel outage of the traditional scheme starts before 30 degrees of the FOV but in the case of the blue color channel outage starts after 50 degrees, as shown in Figure 10. This variation is the cause of severe degradation of the VLC receiver performance when the receiver is shifted from one color channel to another. The performance variation also creates the unfairness situation for the receiver only for using different channel colors. Our proposed scheme makes the outage probability of each color of channel close together and reduces the performance variation of the receiver.

## 5. Conclusions

The performance variation at the receiver is the important issue for visible light multi-color channel communication. Due to the photo sensitivity of the photo-detector the performance is not same, even though same transmitter and receiver are being used. We have proposed a receiver considering the performance variation on the different color of channels according to the VLC band plan. Our proposed scheme has the ability to reduce the performance variation only at the receiver of the visible light communication. Our proposed system considers only three channel colors. It can be extended to all channel colors to enhance the performance in the future. In this paper we propose a receiver structure and prove its functionality mathematically as well as by simulation.

## Figures and Tables

**Table 1.** Basic assumptions.

**Parameter**	**Value**
Photo-detector area, *A*	0.9 (cm^2^)
Transmission coefficient of filter, *T_s_*(*ψ*)	1.0
Concentrator FOV, *ψ_c_*	60 (degree)
Semi-angle at half power, *Φ*_1/2_	15 (degree)
Sensitivity of photo-detector	
On red color at 625 nm	0.43 (A/W)
On green color at 535 nm	0.32 (A/W)
On blue color at 480 nm	0.27 (A/W)
Refractive index of lens	
*n_R_* (Tuning value)	1.00040
*n_G_* (Tuning value)	1.00050
*n_B_* (Tuning value)	1.00075
Bandwidth in terms of wavelength	
*Δλ_R_*	50 (nm)
*Δλ_G_*	50 (nm)
*Δλ_B_*	70 (nm)
